# 5′′-(4-Nitro­benzyl­idene)-7′-(4-nitro­phen­yl)-1′′-methyl-1′,3′,5′,6′,7′,7a′-hexa­hydro­dispiro­[ace­naphthyl­ene-1,5′-pyrrolo­[1,2-*c*][1,3]thia­zole-6′,3′′-piperidine]-2,4′′(1*H*)-dione including an unknown solvate

**DOI:** 10.1107/S1600536813017704

**Published:** 2013-07-06

**Authors:** R. Vishnupriya, J. Suresh, S. Sivakumar, R. Ranjith. Kumar, P. L. Nilantha Lakshman

**Affiliations:** a Department of Physics, The Madura College, Madurai 625 011, India; b Department of Organic Chemistry, School of Chemistry, Madurai Kamaraj University, Madurai 625 021, India; c Department of Food Science and Technology, University of Ruhuna, Mapalana, Kamburupitiya 81100, Sri Lanka

## Abstract

The title compound, C_35_H_28_N_4_O_6_S, crystallizes with two mol­ecules in the asymmetric unit. In both mol­ecules, the piperidine ring adopts a shallow-chair conformation, the thia­zole ring adopts a twisted conformation about the C_m_—N bond (m = methine) and the pyrrole ring adopts an envelope conformation with the C atom shared with the thia­zole ring as the flap. In the crystal, inversion dimers linked by pairs of C—H⋯O inter­actions generate *R*_2_^2^(34) loops for one of the asymmetric mol­ecules. Further C—H⋯O links also involving the other mol­ecule lead to a three-dimesional network. The contribution of the highly disordered solvent to the scattering was removed with SQUEEZE option of *PLATON* [Spek (2009 ▶). *Acta Cryst.* D**65**, 148–155]. The solvent contribution is not included in the reported mol­ecular weight and density.

## Related literature

For a related structure and background to spiro-pyrrolidine compounds, see: Suresh *et al.* (2013 ▶).
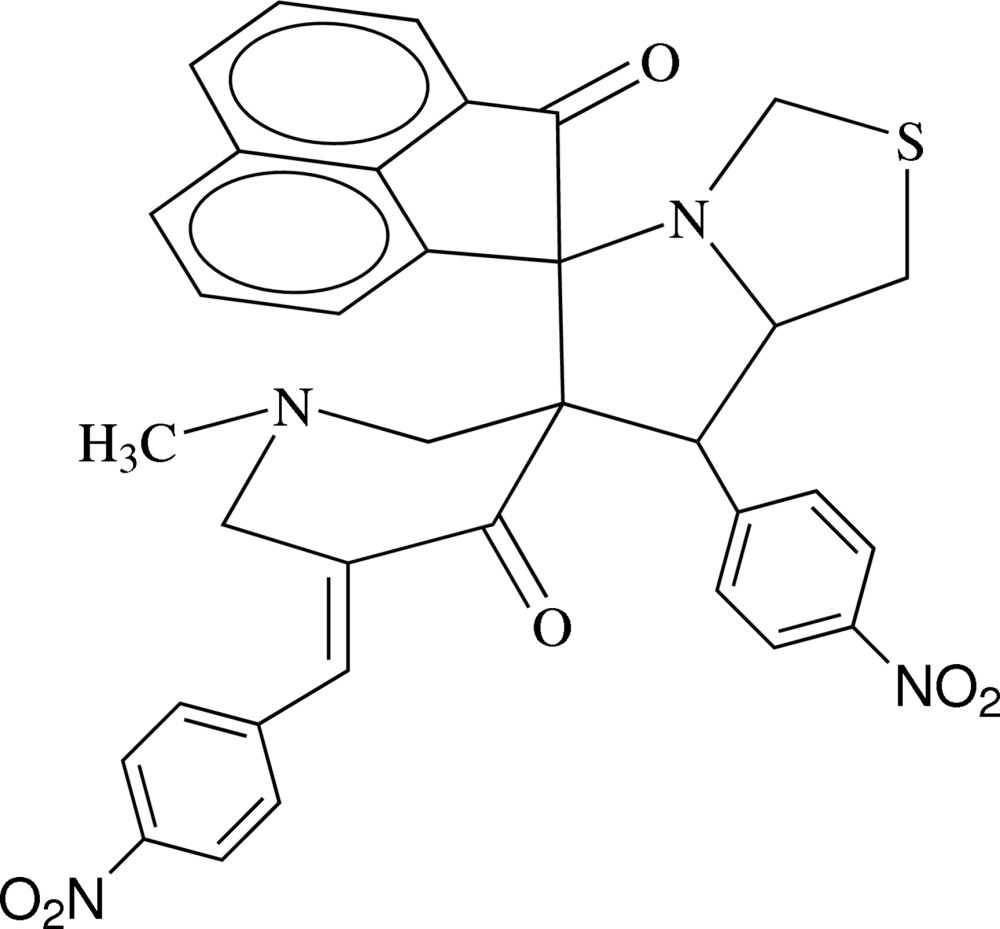


## Experimental

### 

#### Crystal data

C_35_H_28_N_4_O_6_S
*M* *_r_* = 632.67
Triclinic, 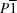

*a* = 11.1240 (5) Å
*b* = 12.6825 (5) Å
*c* = 25.8778 (11) Å
α = 89.823 (4)°
β = 81.185 (2)°
γ = 88.305 (3)°
*V* = 3606.1 (3) Å^3^
*Z* = 4
Mo *K*α radiation
μ = 0.14 mm^−1^
*T* = 293 K
0.30 × 0.27 × 0.25 mm

#### Data collection

Bruker Kappa APEXII diffractometer
Absorption correction: multi-scan (*SADABS*; Sheldrick, 1996 ▶) *T*_min_ = 0.960, *T*_max_ = 0.967
71136 measured reflections
15643 independent reflections
9029 reflections with *I* > 2σ(*I*)
*R*_int_ = 0.040

#### Refinement

*R*[*F* ^2^ > 2σ(*F* ^2^)] = 0.050
*wR*(*F* ^2^) = 0.143
*S* = 1.02
15643 reflections
831 parameters
H-atom parameters constrained
Δρ_max_ = 0.23 e Å^−3^
Δρ_min_ = −0.26 e Å^−3^

### 

Data collection: *APEX2* (Bruker, 2004 ▶); cell refinement: *SAINT* (Bruker, 2004 ▶); data reduction: *SAINT*; program(s) used to solve structure: *SHELXS97* (Sheldrick, 2008 ▶); program(s) used to refine structure: *SHELXL97* (Sheldrick, 2008 ▶); molecular graphics: *PLATON* (Spek, 2009 ▶); software used to prepare material for publication: *SHELXL97*.

## Supplementary Material

Crystal structure: contains datablock(s) global, I. DOI: 10.1107/S1600536813017704/hb7098sup1.cif

Structure factors: contains datablock(s) I. DOI: 10.1107/S1600536813017704/hb7098Isup2.hkl

Additional supplementary materials:  crystallographic information; 3D view; checkCIF report

## Figures and Tables

**Table 1:** Hydrogen-bond geometry (Å, °)

*D*—H⋯*A*	*D*—H	H⋯*A*	*D*⋯*A*	*D*—H⋯*A*
C8*B*—H8*B*⋯O4*A*	0.98	2.47	3.270 (2)	139
C72*A*—H72*A*⋯O4*B*	0.93	2.55	3.461 (2)	167
C76*B*—H76*B*⋯O4*A*	0.93	2.30	3.222 (2)	174
C15*B*—H15*B*⋯O3*B* ^i^	0.93	2.50	3.343 (3)	151

Symmetry code: (i) 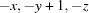.

**Fig. 1.**
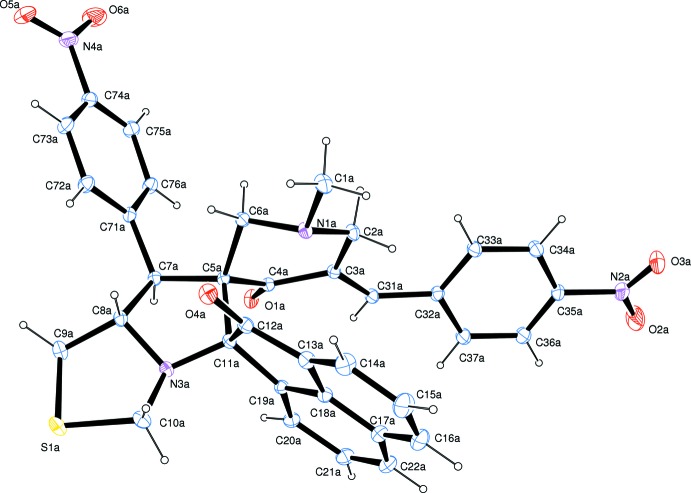
The molecular structure of (IA), showing 10% probability displacement ellipsoids.

**Fig. 2.**
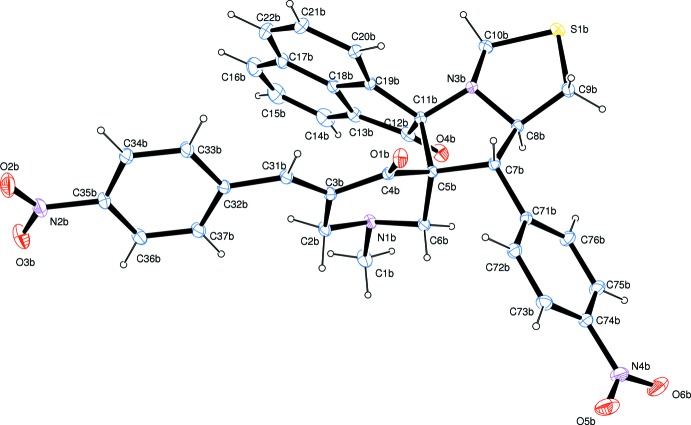
The molecular structure of (IB), showing 10% probability displacement ellipsoids.

**Fig. 3.**
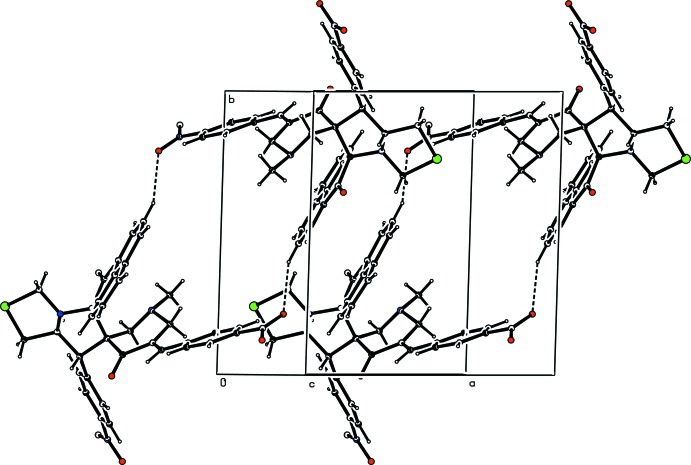
The partial packing diagram of the IB molecule showing the graph set motif R22(34).

## supplementary crystallographic information

### Comment

As part of our ongoing studies of spiro-pyrrolidine compounds (Suresh *et al.*, 2013), we synthesized the title compound, the structure of which is reported here.

The asymmetric unit of the title compound C_35_H_28_N_4_O_6_S, contains two independent molecules (A and B) with almost identical geometry (Figs. 1 and 2). The central piperidine ring adopts a twisted conformation in both the molecules, as evidenced by the puckering parameters q_2_ = 0.5497 (2) Å, θ = 36.97 (2)°, φ = 300.8 (3)° and q_2_ = 0.5441 (17) Å, θ = 143.50 (2)°, φ = 120.4 (3)° for molecule A and B respectively. The 2*H*-acenaphthylen-1-one ring system (C11A—C22A) and (C11B—C22B) is nearly planar, with a maximum displacement of -0.0635 (3) Å and 0.0755 (2) Å for atom C20A and C20B respectively. In the pyrrolo thiazole ring in both the molecules, the pyrrole ring is in the envelope conformation, and thiazole ring is in twisted envelope conformation with C8A and C8B atoms at the flap in both of these envelopes.The sum of the angles at atom N1A and N1B are 336.20 (11)° and 336.36 (2)° in molecule A and B are in accordance with *sp*^3^ -hybridization.The dihedral angle between the nitrophenyl rings in molecule A and B are 76.73 (1)° and 82.93 (1)° respectively these rings are making angles of 44.79 (1)°, 59.27 (1)° and 40.68 (1)°, 57.91 (1)° with the acenaphthene group of molecule A and B respectively.

There are several intramolecular interactions. The C15B—H15B···O3B intermolecular hydrogen bond centrosymmetrically connect two molecules and generate the graph set motif *R*_2_^2^(34) (Fig. 3).

### Experimental

A mixture of 1-methyl-3,5-bis[(*E*)-4-nitrophenylmethylidene]tetrahydro-4(1*H*)-pyridinone (1 mmol), acenaphthenequinone (0.182 g, 1 mmol) and 1,3-thiazolane-4-carboxylic acid (0.133 g, 1 mmol) was dissolved in methanol (10 ml) and refluxed for 30 min. After completion of the reaction as evident from TLC, the mixture was poured into water (50 ml), the precipitated solid was filtered and washed with water (100 ml) to obtain pure product as pale yellow solid. The product was recrystallized from ethyl acetate solution to obtain colourless blocks. Melting point: 230 °C, yield: 94%

### Refinement

Initial structural solution showed co-crystallized solvent molecule for which a suitable model could not be found. Therefore, the data set was treated with SQUEEZE routine of *PLATON* (Spek,2009) to model the electron density in the void region. There is one cavity of 833 Å^3^. The cavity contains approximately 138 electrons which were assigned to the solvent molecule. H atoms were placed at calculated positions and allowed to ride on their carrier atoms with C—H = 0.93–0.98 Å. *U*_iso_ = 1.2*U*_eq_(C) for CH_2_ and CH groups and *U*_iso_ = 1.5*U*_eq_(C) for CH_3_ group.

### Crystal data

**Table:**

C_35_H_28_N_4_O_6_S	*Z* = 4
*M**_r_* = 632.67	*F*(000) = 1320
Triclinic, *P*1	*D*_x_ = 1.165 Mg m^−^^3^
Hall symbol: -P 1	Mo *K*α radiation, λ = 0.71073 Å
*a* = 11.1240 (5) Å	Cell parameters from 2000 reflections
*b* = 12.6825 (5) Å	θ = 2–27°
*c* = 25.8778 (11) Å	µ = 0.14 mm^−^^1^
α = 89.823 (4)°	*T* = 293 K
β = 81.185 (2)°	Block, colourless
γ = 88.305 (3)°	0.30 × 0.27 × 0.25 mm
*V* = 3606.1 (3) Å^3^	

### Data collection

**Table:**

Bruker Kappa APEXII diffractometer	15643 independent reflections
Radiation source: fine-focus sealed tube	9029 reflections with *I* > 2σ(*I*)
Graphite monochromator	*R*_int_ = 0.040
Detector resolution: 0 pixels mm^-1^	θ_max_ = 27.0°, θ_min_ = 1.6°
ω and φ scans	*h* = −14→13
Absorption correction: multi-scan (*SADABS*; Sheldrick, 1996)	*k* = −16→16
*T*_min_ = 0.960, *T*_max_ = 0.967	*l* = −32→31
71136 measured reflections	

### Refinement

**Table:**

Refinement on *F*^2^	Primary atom site location: structure-invariant direct methods
Least-squares matrix: full	Secondary atom site location: difference Fourier map
*R*[*F*^2^ > 2σ(*F*^2^)] = 0.050	Hydrogen site location: inferred from neighbouring sites
*wR*(*F*^2^) = 0.143	H-atom parameters constrained
*S* = 1.02	*w* = 1/[σ^2^(*F*_o_^2^) + (0.0727*P*)^2^] where *P* = (*F*_o_^2^ + 2*F*_c_^2^)/3
15643 reflections	(Δ/σ)_max_ = 0.001
831 parameters	Δρ_max_ = 0.23 e Å^−^^3^
0 restraints	Δρ_min_ = −0.26 e Å^−^^3^

### Special details

**Table:**

Geometry. All e.s.d.'s (except the e.s.d. in the dihedral angle between two l.s. planes) are estimated using the full covariance matrix. The cell e.s.d.'s are taken into account individually in the estimation of e.s.d.'s in distances, angles and torsion angles; correlations between e.s.d.'s in cell parameters are only used when they are defined by crystal symmetry. An approximate (isotropic) treatment of cell e.s.d.'s is used for estimating e.s.d.'s involving l.s. planes.
Refinement. Refinement of *F*^2^ against ALL reflections. The weighted *R*-factor *wR* and goodness of fit *S* are based on *F*^2^, conventional *R*-factors *R* are based on *F*, with *F* set to zero for negative *F*^2^. The threshold expression of *F*^2^ > σ(*F*^2^) is used only for calculating *R*-factors(gt) *etc*. and is not relevant to the choice of reflections for refinement. *R*-factors based on *F*^2^ are statistically about twice as large as those based on *F*, and *R*- factors based on ALL data will be even larger.

### Fractional atomic coordinates and isotropic or equivalent isotropic displacement parameters (Å^2^)

**Table:**

	*x*	*y*	*z*	*U*_iso_*/*U*_eq_	
C1A	0.87585 (18)	0.70954 (17)	0.29382 (7)	0.0604 (6)	
H1A1	0.9184	0.7660	0.3069	0.091*	
H1A2	0.8357	0.7350	0.2657	0.091*	
H1A3	0.9328	0.6536	0.2813	0.091*	
C1B	0.13036 (19)	0.72069 (19)	0.19002 (8)	0.0692 (6)	
H1B1	0.0739	0.7715	0.2084	0.104*	
H1B2	0.1737	0.6843	0.2144	0.104*	
H1B3	0.0868	0.6709	0.1727	0.104*	
C2A	0.84298 (16)	0.63470 (15)	0.37957 (7)	0.0464 (5)	
H2A1	0.8781	0.6947	0.3943	0.056*	
H2A2	0.9088	0.5848	0.3671	0.056*	
C2B	0.15413 (17)	0.82294 (15)	0.11163 (7)	0.0487 (5)	
H2B1	0.0867	0.8669	0.1285	0.058*	
H2B2	0.1208	0.7680	0.0925	0.058*	
C3A	0.75707 (15)	0.58349 (13)	0.42185 (6)	0.0382 (4)	
C3B	0.23505 (15)	0.88878 (13)	0.07375 (6)	0.0397 (4)	
C4A	0.64253 (15)	0.53868 (13)	0.40878 (6)	0.0371 (4)	
C4B	0.34834 (16)	0.93051 (14)	0.08959 (6)	0.0381 (4)	
C5A	0.59835 (15)	0.58108 (13)	0.35993 (6)	0.0351 (4)	
C5B	0.39843 (14)	0.87307 (13)	0.13346 (6)	0.0350 (4)	
C6A	0.71055 (15)	0.59021 (14)	0.31842 (6)	0.0412 (4)	
H6A1	0.7551	0.5231	0.3142	0.049*	
H6A2	0.6867	0.6099	0.2851	0.049*	
C6B	0.29024 (15)	0.84790 (14)	0.17458 (6)	0.0432 (4)	
H6B1	0.3180	0.8168	0.2051	0.052*	
H6B2	0.2432	0.9119	0.1853	0.052*	
C7A	0.49715 (15)	0.51427 (13)	0.34182 (6)	0.0386 (4)	
H7A	0.4543	0.4796	0.3729	0.046*	
C7B	0.49726 (15)	0.93677 (13)	0.15573 (6)	0.0378 (4)	
H7B	0.5383	0.9791	0.1269	0.045*	
C8A	0.41036 (16)	0.59708 (14)	0.32496 (7)	0.0433 (4)	
H8A	0.4449	0.6276	0.2913	0.052*	
C8B	0.58715 (15)	0.85067 (13)	0.16685 (7)	0.0398 (4)	
H8B	0.5541	0.8101	0.1978	0.048*	
C9A	0.27896 (18)	0.57110 (17)	0.32504 (9)	0.0625 (6)	
H9A1	0.2533	0.5181	0.3512	0.075*	
H9A2	0.2667	0.5451	0.2911	0.075*	
C9B	0.71781 (16)	0.87805 (15)	0.16871 (8)	0.0510 (5)	
H9B1	0.7292	0.8951	0.2041	0.061*	
H9B2	0.7411	0.9378	0.1463	0.061*	
C10A	0.32372 (18)	0.75971 (16)	0.36064 (9)	0.0593 (5)	
H10A	0.3589	0.8089	0.3342	0.071*	
H10B	0.2997	0.7973	0.3934	0.071*	
C10B	0.68135 (17)	0.70191 (16)	0.11956 (8)	0.0552 (5)	
H10C	0.7075	0.6754	0.0844	0.066*	
H10D	0.6481	0.6445	0.1416	0.066*	
C11A	0.53387 (15)	0.69461 (13)	0.37313 (6)	0.0379 (4)	
C11B	0.46986 (15)	0.76761 (13)	0.11067 (6)	0.0369 (4)	
C12A	0.59294 (16)	0.78160 (14)	0.33547 (7)	0.0444 (4)	
C12B	0.41528 (17)	0.66522 (15)	0.13826 (8)	0.0506 (5)	
C13A	0.63341 (17)	0.86576 (14)	0.36697 (8)	0.0493 (5)	
C13B	0.37407 (18)	0.59876 (15)	0.09764 (9)	0.0559 (5)	
C14A	0.6892 (2)	0.95854 (16)	0.35387 (10)	0.0683 (6)	
H14A	0.7103	0.9782	0.3191	0.082*	
C14B	0.3186 (2)	0.50281 (17)	0.09947 (12)	0.0806 (7)	
H14B	0.2985	0.4670	0.1309	0.097*	
C15A	0.7135 (2)	1.02295 (18)	0.39437 (12)	0.0854 (8)	
H15A	0.7519	1.0862	0.3861	0.102*	
C15B	0.2937 (3)	0.4611 (2)	0.05219 (15)	0.0987 (10)	
H15B	0.2565	0.3963	0.0528	0.118*	
C16A	0.6825 (2)	0.99621 (18)	0.44620 (11)	0.0794 (7)	
H16A	0.6998	1.0417	0.4720	0.095*	
C16B	0.3215 (2)	0.5114 (2)	0.00561 (14)	0.0936 (9)	
H16B	0.3016	0.4807	−0.0244	0.112*	
C17A	0.62507 (19)	0.90061 (17)	0.46055 (9)	0.0609 (6)	
C17B	0.37991 (19)	0.60908 (17)	0.00180 (10)	0.0653 (6)	
C18A	0.60165 (16)	0.83738 (14)	0.41950 (7)	0.0458 (4)	
C18B	0.40406 (16)	0.64970 (14)	0.04985 (8)	0.0483 (5)	
C19A	0.54073 (16)	0.74210 (13)	0.42631 (7)	0.0399 (4)	
C19B	0.46462 (15)	0.74379 (13)	0.05355 (7)	0.0393 (4)	
C20A	0.49753 (17)	0.71036 (15)	0.47580 (7)	0.0497 (5)	
H20A	0.4523	0.6498	0.4816	0.060*	
C20B	0.50629 (17)	0.79583 (15)	0.00874 (7)	0.0481 (5)	
H20B	0.5517	0.8560	0.0097	0.058*	
C21A	0.5228 (2)	0.77132 (18)	0.51814 (8)	0.0652 (6)	
H21A	0.4963	0.7482	0.5520	0.078*	
C21B	0.4802 (2)	0.75799 (19)	−0.03908 (8)	0.0661 (6)	
H21B	0.5063	0.7956	−0.0694	0.079*	
C22A	0.5843 (2)	0.86216 (19)	0.51140 (9)	0.0711 (7)	
H22A	0.5998	0.8995	0.5404	0.085*	
C31A	0.77292 (16)	0.57557 (14)	0.47200 (7)	0.0432 (4)	
H31A	0.7110	0.5420	0.4936	0.052*	
C31B	0.21664 (16)	0.91406 (14)	0.02528 (7)	0.0451 (4)	
H31B	0.2766	0.9558	0.0073	0.054*	
C32A	0.87179 (17)	0.61111 (14)	0.49840 (7)	0.0440 (4)	
C32B	0.12066 (16)	0.88869 (14)	−0.00463 (7)	0.0439 (4)	
C33A	0.98950 (17)	0.63058 (16)	0.47390 (7)	0.0524 (5)	
H33A	1.0085	0.6237	0.4378	0.063*	
C33B	0.14564 (18)	0.90447 (18)	−0.05837 (7)	0.0582 (5)	
H33B	0.2224	0.9267	−0.0729	0.070*	
C34A	1.07798 (18)	0.65980 (16)	0.50214 (8)	0.0558 (5)	
H34A	1.1559	0.6739	0.4853	0.067*	
C34B	0.06142 (19)	0.88838 (19)	−0.09054 (8)	0.0636 (6)	
H34B	0.0796	0.9003	−0.1263	0.076*	
C35A	1.05014 (19)	0.66777 (16)	0.55505 (8)	0.0568 (5)	
C35B	−0.05076 (18)	0.85414 (16)	−0.06860 (8)	0.0536 (5)	
C36A	0.9368 (2)	0.6493 (2)	0.58089 (8)	0.0724 (7)	
H36A	0.9200	0.6551	0.6171	0.087*	
C36B	−0.07985 (18)	0.83640 (16)	−0.01624 (8)	0.0577 (5)	
H36B	−0.1563	0.8127	−0.0023	0.069*	
C37A	0.84723 (19)	0.62175 (18)	0.55242 (8)	0.0632 (6)	
H37A	0.7691	0.6101	0.5697	0.076*	
C37B	0.00563 (18)	0.85414 (16)	0.01576 (8)	0.0550 (5)	
H37B	−0.0139	0.8428	0.0516	0.066*	
C71A	0.54515 (16)	0.42844 (14)	0.30287 (7)	0.0413 (4)	
C71B	0.44803 (15)	1.01252 (13)	0.19966 (6)	0.0394 (4)	
C72A	0.53556 (19)	0.43517 (16)	0.25039 (7)	0.0550 (5)	
H72A	0.4968	0.4938	0.2379	0.066*	
C72B	0.38829 (17)	1.10487 (15)	0.18855 (8)	0.0501 (5)	
H72B	0.3778	1.1193	0.1542	0.060*	
C73A	0.5829 (2)	0.35578 (18)	0.21644 (8)	0.0621 (6)	
H73A	0.5751	0.3603	0.1812	0.074*	
C73B	0.34393 (18)	1.17594 (16)	0.22747 (8)	0.0562 (5)	
H73B	0.3033	1.2377	0.2197	0.067*	
C74A	0.64099 (18)	0.27107 (16)	0.23445 (8)	0.0566 (5)	
C74B	0.36056 (17)	1.15436 (16)	0.27766 (8)	0.0527 (5)	
C75A	0.65213 (19)	0.26152 (15)	0.28631 (8)	0.0586 (5)	
H75A	0.6922	0.2031	0.2983	0.070*	
C75B	0.42004 (19)	1.06533 (17)	0.28988 (8)	0.0599 (6)	
H75B	0.4314	1.0523	0.3242	0.072*	
C76A	0.60285 (18)	0.34007 (14)	0.32015 (8)	0.0513 (5)	
H76A	0.6084	0.3337	0.3555	0.062*	
C76B	0.46353 (18)	0.99430 (16)	0.25079 (7)	0.0537 (5)	
H76B	0.5041	0.9329	0.2591	0.064*	
C22B	0.4187 (2)	0.6692 (2)	−0.04262 (9)	0.0768 (7)	
H22B	0.4018	0.6476	−0.0750	0.092*	
N1A	0.78620 (13)	0.67026 (11)	0.33553 (5)	0.0418 (4)	
N1B	0.21612 (13)	0.77429 (12)	0.15153 (5)	0.0450 (4)	
N2A	1.1455 (2)	0.69628 (17)	0.58551 (10)	0.0790 (6)	
N2B	−0.14135 (18)	0.83732 (17)	−0.10316 (9)	0.0706 (5)	
N3A	0.40829 (13)	0.67384 (11)	0.36675 (6)	0.0426 (4)	
N3B	0.59309 (12)	0.78733 (11)	0.11969 (5)	0.0399 (3)	
N4A	0.69134 (19)	0.18757 (18)	0.19817 (10)	0.0772 (6)	
N4B	0.31700 (18)	1.23068 (17)	0.31917 (9)	0.0731 (6)	
O1A	0.58766 (12)	0.47261 (10)	0.43609 (5)	0.0520 (3)	
O1B	0.39758 (12)	1.00630 (10)	0.06827 (5)	0.0541 (3)	
O2A	1.12997 (19)	0.6756 (2)	0.63191 (9)	0.1399 (10)	
O2B	−0.12558 (16)	0.87893 (19)	−0.14615 (7)	0.1109 (7)	
O3A	1.23600 (18)	0.73573 (15)	0.56316 (9)	0.1026 (6)	
O3B	−0.22908 (17)	0.78737 (15)	−0.08692 (8)	0.0953 (6)	
O4A	0.59126 (12)	0.78238 (10)	0.28855 (5)	0.0558 (4)	
O4B	0.42138 (13)	0.64232 (11)	0.18328 (6)	0.0669 (4)	
O5A	0.66833 (18)	0.18978 (16)	0.15397 (8)	0.1059 (7)	
O5B	0.25968 (19)	1.30840 (16)	0.30738 (8)	0.1013 (6)	
O6A	0.7540 (2)	0.11787 (16)	0.21417 (8)	0.1069 (7)	
O6B	0.33981 (19)	1.21481 (17)	0.36269 (8)	0.1094 (7)	
S1A	0.19481 (5)	0.69505 (5)	0.34043 (2)	0.07070 (18)	
S1B	0.80665 (5)	0.76056 (5)	0.14550 (2)	0.06367 (17)	

### Atomic displacement parameters (Å^2^)

**Table:**

	*U*^11^	*U*^22^	*U*^33^	*U*^12^	*U*^13^	*U*^23^
C1A	0.0512 (12)	0.0823 (15)	0.0464 (12)	−0.0176 (11)	−0.0003 (9)	0.0178 (11)
C1B	0.0558 (13)	0.0995 (18)	0.0534 (13)	−0.0258 (12)	−0.0078 (10)	0.0315 (12)
C2A	0.0404 (11)	0.0578 (12)	0.0425 (11)	−0.0058 (9)	−0.0105 (8)	0.0085 (9)
C2B	0.0440 (11)	0.0610 (12)	0.0430 (11)	−0.0053 (9)	−0.0127 (9)	0.0120 (9)
C3A	0.0379 (10)	0.0409 (10)	0.0363 (10)	0.0025 (8)	−0.0085 (8)	0.0038 (8)
C3B	0.0401 (10)	0.0432 (10)	0.0366 (10)	0.0035 (8)	−0.0089 (8)	0.0039 (8)
C4A	0.0400 (10)	0.0371 (9)	0.0339 (9)	−0.0003 (8)	−0.0053 (8)	0.0027 (8)
C4B	0.0433 (10)	0.0392 (10)	0.0314 (9)	0.0012 (8)	−0.0050 (8)	0.0013 (8)
C5A	0.0379 (10)	0.0389 (9)	0.0290 (9)	−0.0024 (7)	−0.0065 (7)	0.0047 (7)
C5B	0.0351 (9)	0.0396 (9)	0.0303 (9)	−0.0018 (7)	−0.0050 (7)	0.0058 (7)
C6A	0.0391 (10)	0.0503 (11)	0.0337 (9)	−0.0002 (8)	−0.0050 (8)	0.0032 (8)
C6B	0.0391 (10)	0.0572 (11)	0.0336 (10)	−0.0014 (9)	−0.0064 (8)	0.0074 (8)
C7A	0.0393 (10)	0.0425 (10)	0.0343 (9)	−0.0043 (8)	−0.0062 (8)	0.0036 (8)
C7B	0.0414 (10)	0.0409 (10)	0.0318 (9)	−0.0030 (8)	−0.0074 (7)	0.0034 (7)
C8A	0.0404 (11)	0.0496 (11)	0.0417 (10)	−0.0037 (8)	−0.0117 (8)	0.0051 (8)
C8B	0.0392 (10)	0.0453 (10)	0.0353 (10)	−0.0024 (8)	−0.0072 (8)	0.0032 (8)
C9A	0.0472 (12)	0.0723 (14)	0.0723 (14)	−0.0045 (10)	−0.0222 (11)	0.0021 (11)
C9B	0.0445 (11)	0.0598 (12)	0.0513 (12)	−0.0056 (9)	−0.0146 (9)	0.0001 (9)
C10A	0.0468 (12)	0.0631 (13)	0.0682 (14)	0.0092 (10)	−0.0111 (10)	0.0021 (11)
C10B	0.0433 (11)	0.0595 (12)	0.0629 (13)	0.0071 (10)	−0.0095 (10)	−0.0095 (10)
C11A	0.0366 (10)	0.0409 (10)	0.0367 (10)	−0.0003 (8)	−0.0073 (7)	0.0050 (8)
C11B	0.0381 (10)	0.0370 (9)	0.0366 (9)	−0.0053 (8)	−0.0080 (8)	0.0060 (7)
C12A	0.0412 (11)	0.0474 (11)	0.0447 (12)	0.0038 (8)	−0.0081 (8)	0.0092 (9)
C12B	0.0430 (11)	0.0460 (11)	0.0629 (14)	−0.0001 (9)	−0.0088 (10)	0.0152 (10)
C13A	0.0487 (12)	0.0425 (11)	0.0564 (13)	−0.0032 (9)	−0.0067 (9)	0.0069 (9)
C13B	0.0471 (12)	0.0384 (11)	0.0829 (16)	−0.0047 (9)	−0.0116 (11)	0.0029 (10)
C14A	0.0778 (16)	0.0484 (12)	0.0782 (16)	−0.0148 (11)	−0.0080 (12)	0.0108 (11)
C14B	0.0727 (17)	0.0467 (13)	0.123 (2)	−0.0125 (12)	−0.0141 (15)	0.0117 (14)
C15A	0.0949 (19)	0.0524 (14)	0.109 (2)	−0.0266 (13)	−0.0130 (16)	−0.0023 (14)
C15B	0.085 (2)	0.0528 (15)	0.163 (3)	−0.0200 (14)	−0.031 (2)	−0.0179 (19)
C16A	0.0856 (18)	0.0629 (15)	0.091 (2)	−0.0151 (13)	−0.0153 (15)	−0.0229 (14)
C16B	0.0790 (19)	0.0742 (18)	0.133 (3)	−0.0129 (15)	−0.0315 (18)	−0.0397 (18)
C17A	0.0572 (13)	0.0565 (13)	0.0689 (15)	−0.0021 (10)	−0.0087 (11)	−0.0160 (11)
C17B	0.0527 (13)	0.0634 (14)	0.0834 (17)	−0.0029 (11)	−0.0206 (12)	−0.0277 (13)
C18A	0.0427 (11)	0.0431 (10)	0.0515 (12)	0.0007 (8)	−0.0078 (9)	−0.0033 (9)
C18B	0.0404 (11)	0.0436 (11)	0.0617 (13)	−0.0005 (9)	−0.0103 (9)	−0.0068 (10)
C19A	0.0397 (10)	0.0420 (10)	0.0378 (10)	0.0029 (8)	−0.0061 (8)	0.0010 (8)
C19B	0.0380 (10)	0.0389 (10)	0.0418 (10)	−0.0014 (8)	−0.0084 (8)	−0.0016 (8)
C20A	0.0512 (12)	0.0538 (11)	0.0420 (11)	−0.0014 (9)	−0.0005 (9)	0.0003 (9)
C20B	0.0468 (11)	0.0559 (12)	0.0409 (11)	−0.0027 (9)	−0.0039 (9)	−0.0030 (9)
C21A	0.0663 (15)	0.0847 (16)	0.0420 (12)	0.0004 (13)	−0.0002 (10)	−0.0082 (11)
C21B	0.0675 (15)	0.0894 (17)	0.0411 (12)	−0.0001 (13)	−0.0079 (10)	−0.0089 (11)
C22A	0.0726 (16)	0.0831 (17)	0.0568 (15)	−0.0037 (13)	−0.0064 (12)	−0.0263 (13)
C31A	0.0410 (11)	0.0501 (11)	0.0389 (10)	−0.0006 (8)	−0.0077 (8)	0.0040 (8)
C31B	0.0430 (11)	0.0529 (11)	0.0395 (10)	0.0005 (9)	−0.0075 (8)	0.0064 (8)
C32A	0.0478 (11)	0.0491 (11)	0.0373 (10)	0.0015 (9)	−0.0135 (8)	0.0010 (8)
C32B	0.0410 (11)	0.0545 (11)	0.0369 (10)	0.0035 (9)	−0.0090 (8)	0.0060 (8)
C33A	0.0454 (12)	0.0690 (13)	0.0437 (11)	−0.0005 (10)	−0.0102 (9)	−0.0001 (10)
C33B	0.0441 (12)	0.0905 (16)	0.0409 (11)	−0.0071 (11)	−0.0086 (9)	0.0071 (11)
C34A	0.0442 (12)	0.0647 (13)	0.0597 (14)	−0.0019 (10)	−0.0121 (10)	0.0016 (10)
C34B	0.0500 (13)	0.1034 (18)	0.0383 (11)	−0.0004 (12)	−0.0102 (10)	0.0022 (11)
C35A	0.0483 (13)	0.0630 (13)	0.0634 (14)	0.0015 (10)	−0.0225 (11)	−0.0150 (10)
C35B	0.0484 (12)	0.0630 (13)	0.0533 (13)	0.0027 (10)	−0.0213 (10)	0.0023 (10)
C36A	0.0607 (15)	0.1137 (19)	0.0450 (12)	0.0002 (13)	−0.0145 (11)	−0.0198 (13)
C36B	0.0415 (12)	0.0729 (14)	0.0595 (14)	−0.0029 (10)	−0.0105 (10)	0.0166 (11)
C37A	0.0496 (13)	0.0968 (17)	0.0439 (12)	−0.0055 (12)	−0.0083 (10)	−0.0068 (11)
C37B	0.0474 (12)	0.0749 (14)	0.0428 (11)	0.0025 (10)	−0.0081 (9)	0.0130 (10)
C71A	0.0421 (10)	0.0438 (10)	0.0394 (10)	−0.0080 (8)	−0.0098 (8)	0.0003 (8)
C71B	0.0396 (10)	0.0425 (10)	0.0370 (10)	−0.0041 (8)	−0.0078 (8)	0.0012 (8)
C72A	0.0649 (13)	0.0593 (12)	0.0424 (11)	−0.0002 (10)	−0.0131 (10)	0.0013 (9)
C72B	0.0521 (12)	0.0522 (12)	0.0473 (11)	0.0052 (9)	−0.0134 (9)	−0.0006 (9)
C73A	0.0674 (15)	0.0776 (15)	0.0418 (12)	−0.0062 (12)	−0.0091 (10)	−0.0118 (11)
C73B	0.0503 (12)	0.0494 (12)	0.0681 (14)	0.0073 (9)	−0.0082 (10)	−0.0041 (10)
C74A	0.0477 (12)	0.0579 (13)	0.0627 (14)	−0.0091 (10)	−0.0016 (10)	−0.0169 (11)
C74B	0.0458 (12)	0.0564 (12)	0.0545 (13)	−0.0036 (10)	−0.0027 (9)	−0.0165 (10)
C75A	0.0598 (13)	0.0470 (12)	0.0700 (15)	0.0019 (10)	−0.0139 (11)	−0.0072 (10)
C75B	0.0687 (14)	0.0718 (15)	0.0388 (11)	0.0062 (12)	−0.0089 (10)	−0.0075 (10)
C76A	0.0610 (13)	0.0492 (11)	0.0461 (11)	−0.0011 (10)	−0.0155 (9)	−0.0014 (9)
C76B	0.0666 (14)	0.0543 (12)	0.0407 (11)	0.0096 (10)	−0.0114 (9)	−0.0024 (9)
C22B	0.0753 (17)	0.103 (2)	0.0538 (15)	0.0009 (15)	−0.0166 (12)	−0.0275 (14)
N1A	0.0395 (8)	0.0510 (9)	0.0350 (8)	−0.0079 (7)	−0.0047 (7)	0.0110 (7)
N1B	0.0397 (9)	0.0588 (10)	0.0371 (8)	−0.0105 (7)	−0.0064 (7)	0.0152 (7)
N2A	0.0638 (14)	0.0927 (15)	0.0870 (17)	0.0066 (12)	−0.0328 (13)	−0.0310 (13)
N2B	0.0539 (12)	0.0891 (14)	0.0738 (14)	0.0004 (11)	−0.0263 (11)	0.0032 (11)
N3A	0.0387 (9)	0.0451 (9)	0.0452 (9)	0.0016 (7)	−0.0106 (7)	0.0019 (7)
N3B	0.0362 (8)	0.0457 (8)	0.0384 (8)	0.0005 (7)	−0.0071 (6)	−0.0036 (7)
N4A	0.0627 (14)	0.0795 (15)	0.0840 (16)	−0.0135 (11)	0.0084 (11)	−0.0309 (13)
N4B	0.0649 (13)	0.0738 (14)	0.0754 (15)	−0.0028 (11)	0.0064 (11)	−0.0255 (12)
O1A	0.0633 (9)	0.0539 (8)	0.0423 (7)	−0.0178 (7)	−0.0164 (6)	0.0154 (6)
O1B	0.0700 (9)	0.0492 (8)	0.0473 (8)	−0.0158 (7)	−0.0205 (7)	0.0167 (6)
O2A	0.0940 (15)	0.256 (3)	0.0801 (14)	−0.0194 (17)	−0.0434 (12)	−0.0372 (17)
O2B	0.0825 (13)	0.193 (2)	0.0666 (12)	−0.0259 (13)	−0.0385 (10)	0.0241 (13)
O3A	0.0700 (13)	0.1102 (15)	0.1352 (17)	−0.0202 (11)	−0.0359 (12)	−0.0274 (13)
O3B	0.0745 (12)	0.1022 (14)	0.1211 (15)	−0.0273 (11)	−0.0493 (11)	0.0252 (11)
O4A	0.0629 (9)	0.0641 (9)	0.0424 (8)	−0.0019 (7)	−0.0142 (6)	0.0193 (6)
O4B	0.0657 (10)	0.0726 (10)	0.0646 (10)	−0.0073 (8)	−0.0165 (8)	0.0375 (8)
O5A	0.1053 (15)	0.1330 (17)	0.0745 (12)	−0.0017 (12)	0.0022 (11)	−0.0495 (12)
O5B	0.1089 (15)	0.0783 (12)	0.1069 (14)	0.0171 (11)	0.0114 (11)	−0.0293 (11)
O6A	0.1124 (16)	0.0786 (13)	0.1235 (17)	0.0196 (12)	−0.0020 (13)	−0.0353 (12)
O6B	0.1292 (17)	0.1325 (17)	0.0642 (12)	0.0185 (13)	−0.0105 (11)	−0.0436 (12)
S1A	0.0415 (3)	0.0895 (4)	0.0832 (4)	0.0091 (3)	−0.0184 (3)	−0.0034 (3)
S1B	0.0408 (3)	0.0819 (4)	0.0697 (4)	0.0081 (3)	−0.0144 (3)	−0.0131 (3)

### Geometric parameters (Å, º)

**Table:**

C1A—N1A	1.451 (2)	C15A—H15A	0.9300
C1A—H1A1	0.9600	C15B—C16B	1.359 (4)
C1A—H1A2	0.9600	C15B—H15B	0.9300
C1A—H1A3	0.9600	C16A—C17A	1.410 (3)
C1B—N1B	1.452 (2)	C16A—H16A	0.9300
C1B—H1B1	0.9600	C16B—C17B	1.413 (3)
C1B—H1B2	0.9600	C16B—H16B	0.9300
C1B—H1B3	0.9600	C17A—C18A	1.394 (3)
C2A—N1A	1.450 (2)	C17A—C22A	1.415 (3)
C2A—C3A	1.498 (2)	C17B—C22B	1.397 (3)
C2A—H2A1	0.9700	C17B—C18B	1.414 (3)
C2A—H2A2	0.9700	C18A—C19A	1.400 (2)
C2B—N1B	1.453 (2)	C18B—C19B	1.398 (2)
C2B—C3B	1.496 (2)	C19A—C20A	1.361 (2)
C2B—H2B1	0.9700	C19B—C20B	1.358 (2)
C2B—H2B2	0.9700	C20A—C21A	1.411 (3)
C3A—C31A	1.339 (2)	C20A—H20A	0.9300
C3A—C4A	1.496 (2)	C20B—C21B	1.403 (3)
C3B—C31B	1.338 (2)	C20B—H20B	0.9300
C3B—C4B	1.495 (2)	C21A—C22A	1.354 (3)
C4A—O1A	1.2119 (19)	C21A—H21A	0.9300
C4A—C5A	1.516 (2)	C21B—C22B	1.346 (3)
C4B—O1B	1.210 (2)	C21B—H21B	0.9300
C4B—C5B	1.515 (2)	C22A—H22A	0.9300
C5A—C6A	1.524 (2)	C31A—C32A	1.463 (2)
C5A—C7A	1.557 (2)	C31A—H31A	0.9300
C5A—C11A	1.604 (2)	C31B—C32B	1.455 (2)
C5B—C6B	1.520 (2)	C31B—H31B	0.9300
C5B—C7B	1.563 (2)	C32A—C37A	1.389 (3)
C5B—C11B	1.602 (2)	C32A—C33A	1.393 (3)
C6A—N1A	1.450 (2)	C32B—C37B	1.390 (3)
C6A—H6A1	0.9700	C32B—C33B	1.391 (3)
C6A—H6A2	0.9700	C33A—C34A	1.372 (3)
C6B—N1B	1.451 (2)	C33A—H33A	0.9300
C6B—H6B1	0.9700	C33B—C34B	1.365 (3)
C6B—H6B2	0.9700	C33B—H33B	0.9300
C7A—C8A	1.514 (2)	C34A—C35A	1.360 (3)
C7A—C71A	1.515 (2)	C34A—H34A	0.9300
C7A—H7A	0.9800	C34B—C35B	1.372 (3)
C7B—C8B	1.516 (2)	C34B—H34B	0.9300
C7B—C71B	1.517 (2)	C35A—C36A	1.360 (3)
C7B—H7B	0.9800	C35A—N2A	1.469 (3)
C8A—N3A	1.454 (2)	C35B—C36B	1.364 (3)
C8A—C9A	1.507 (3)	C35B—N2B	1.467 (3)
C8A—H8A	0.9800	C36A—C37A	1.380 (3)
C8B—N3B	1.455 (2)	C36A—H36A	0.9300
C8B—C9B	1.512 (2)	C36B—C37B	1.377 (3)
C8B—H8B	0.9800	C36B—H36B	0.9300
C9A—S1A	1.819 (2)	C37A—H37A	0.9300
C9A—H9A1	0.9700	C37B—H37B	0.9300
C9A—H9A2	0.9700	C71A—C72A	1.381 (2)
C9B—S1B	1.815 (2)	C71A—C76A	1.382 (2)
C9B—H9B1	0.9700	C71B—C76B	1.378 (2)
C9B—H9B2	0.9700	C71B—C72B	1.381 (2)
C10A—N3A	1.442 (2)	C72A—C73A	1.377 (3)
C10A—S1A	1.818 (2)	C72A—H72A	0.9300
C10A—H10A	0.9700	C72B—C73B	1.377 (3)
C10A—H10B	0.9700	C72B—H72B	0.9300
C10B—N3B	1.440 (2)	C73A—C74A	1.355 (3)
C10B—S1B	1.8160 (19)	C73A—H73A	0.9300
C10B—H10C	0.9700	C73B—C74B	1.365 (3)
C10B—H10D	0.9700	C73B—H73B	0.9300
C11A—N3A	1.463 (2)	C74A—C75A	1.371 (3)
C11A—C19A	1.517 (2)	C74A—N4A	1.458 (3)
C11A—C12A	1.562 (2)	C74B—C75B	1.353 (3)
C11B—N3B	1.454 (2)	C74B—N4B	1.465 (3)
C11B—C19B	1.520 (2)	C75A—C76A	1.373 (3)
C11B—C12B	1.572 (2)	C75A—H75A	0.9300
C12A—O4A	1.217 (2)	C75B—C76B	1.378 (3)
C12A—C13A	1.467 (3)	C75B—H75B	0.9300
C12B—O4B	1.211 (2)	C76A—H76A	0.9300
C12B—C13B	1.484 (3)	C76B—H76B	0.9300
C13A—C14A	1.364 (3)	C22B—H22B	0.9300
C13A—C18A	1.400 (3)	N2A—O3A	1.203 (3)
C13B—C14B	1.378 (3)	N2A—O2A	1.215 (3)
C13B—C18B	1.392 (3)	N2B—O3B	1.200 (2)
C14A—C15A	1.394 (3)	N2B—O2B	1.220 (2)
C14A—H14A	0.9300	N4A—O5A	1.210 (3)
C14B—C15B	1.404 (4)	N4A—O6A	1.220 (3)
C14B—H14B	0.9300	N4B—O6B	1.207 (3)
C15A—C16A	1.377 (3)	N4B—O5B	1.221 (3)
			
N1A—C1A—H1A1	109.5	C15A—C16A—H16A	119.6
N1A—C1A—H1A2	109.5	C17A—C16A—H16A	119.6
H1A1—C1A—H1A2	109.5	C15B—C16B—C17B	121.5 (3)
N1A—C1A—H1A3	109.5	C15B—C16B—H16B	119.2
H1A1—C1A—H1A3	109.5	C17B—C16B—H16B	119.2
H1A2—C1A—H1A3	109.5	C18A—C17A—C16A	116.0 (2)
N1B—C1B—H1B1	109.5	C18A—C17A—C22A	115.68 (19)
N1B—C1B—H1B2	109.5	C16A—C17A—C22A	128.3 (2)
H1B1—C1B—H1B2	109.5	C22B—C17B—C16B	129.0 (2)
N1B—C1B—H1B3	109.5	C22B—C17B—C18B	116.23 (19)
H1B1—C1B—H1B3	109.5	C16B—C17B—C18B	114.7 (3)
H1B2—C1B—H1B3	109.5	C17A—C18A—C13A	122.60 (18)
N1A—C2A—C3A	113.67 (14)	C17A—C18A—C19A	123.98 (19)
N1A—C2A—H2A1	108.8	C13A—C18A—C19A	113.37 (16)
C3A—C2A—H2A1	108.8	C13B—C18B—C19B	113.45 (17)
N1A—C2A—H2A2	108.8	C13B—C18B—C17B	123.68 (19)
C3A—C2A—H2A2	108.8	C19B—C18B—C17B	122.8 (2)
H2A1—C2A—H2A2	107.7	C20A—C19A—C18A	118.58 (17)
N1B—C2B—C3B	113.39 (14)	C20A—C19A—C11A	132.23 (16)
N1B—C2B—H2B1	108.9	C18A—C19A—C11A	109.16 (15)
C3B—C2B—H2B1	108.9	C20B—C19B—C18B	118.34 (17)
N1B—C2B—H2B2	108.9	C20B—C19B—C11B	132.19 (15)
C3B—C2B—H2B2	108.9	C18B—C19B—C11B	109.47 (15)
H2B1—C2B—H2B2	107.7	C19A—C20A—C21A	118.58 (19)
C31A—C3A—C4A	115.76 (15)	C19A—C20A—H20A	120.7
C31A—C3A—C2A	125.07 (16)	C21A—C20A—H20A	120.7
C4A—C3A—C2A	119.16 (14)	C19B—C20B—C21B	119.37 (18)
C31B—C3B—C4B	115.16 (16)	C19B—C20B—H20B	120.3
C31B—C3B—C2B	125.78 (16)	C21B—C20B—H20B	120.3
C4B—C3B—C2B	119.06 (14)	C22A—C21A—C20A	122.6 (2)
O1A—C4A—C3A	121.60 (15)	C22A—C21A—H21A	118.7
O1A—C4A—C5A	121.95 (15)	C20A—C21A—H21A	118.7
C3A—C4A—C5A	116.45 (14)	C22B—C21B—C20B	122.4 (2)
O1B—C4B—C3B	121.51 (15)	C22B—C21B—H21B	118.8
O1B—C4B—C5B	121.76 (15)	C20B—C21B—H21B	118.8
C3B—C4B—C5B	116.73 (15)	C21A—C22A—C17A	120.5 (2)
C4A—C5A—C6A	106.64 (13)	C21A—C22A—H22A	119.8
C4A—C5A—C7A	113.46 (13)	C17A—C22A—H22A	119.8
C6A—C5A—C7A	113.25 (13)	C3A—C31A—C32A	130.52 (17)
C4A—C5A—C11A	108.48 (12)	C3A—C31A—H31A	114.7
C6A—C5A—C11A	110.76 (13)	C32A—C31A—H31A	114.7
C7A—C5A—C11A	104.21 (12)	C3B—C31B—C32B	132.21 (18)
C4B—C5B—C6B	106.88 (13)	C3B—C31B—H31B	113.9
C4B—C5B—C7B	112.59 (13)	C32B—C31B—H31B	113.9
C6B—C5B—C7B	113.10 (13)	C37A—C32A—C33A	117.70 (16)
C4B—C5B—C11B	109.06 (13)	C37A—C32A—C31A	117.09 (17)
C6B—C5B—C11B	111.25 (13)	C33A—C32A—C31A	125.14 (16)
C7B—C5B—C11B	103.97 (12)	C37B—C32B—C33B	117.44 (17)
N1A—C6A—C5A	107.80 (13)	C37B—C32B—C31B	126.05 (16)
N1A—C6A—H6A1	110.1	C33B—C32B—C31B	116.47 (17)
C5A—C6A—H6A1	110.1	C34A—C33A—C32A	121.15 (18)
N1A—C6A—H6A2	110.1	C34A—C33A—H33A	119.4
C5A—C6A—H6A2	110.1	C32A—C33A—H33A	119.4
H6A1—C6A—H6A2	108.5	C34B—C33B—C32B	122.27 (19)
N1B—C6B—C5B	107.67 (13)	C34B—C33B—H33B	118.9
N1B—C6B—H6B1	110.2	C32B—C33B—H33B	118.9
C5B—C6B—H6B1	110.2	C35A—C34A—C33A	119.05 (19)
N1B—C6B—H6B2	110.2	C35A—C34A—H34A	120.5
C5B—C6B—H6B2	110.2	C33A—C34A—H34A	120.5
H6B1—C6B—H6B2	108.5	C33B—C34B—C35B	118.15 (18)
C8A—C7A—C71A	117.52 (14)	C33B—C34B—H34B	120.9
C8A—C7A—C5A	103.12 (13)	C35B—C34B—H34B	120.9
C71A—C7A—C5A	113.92 (14)	C36A—C35A—C34A	122.13 (18)
C8A—C7A—H7A	107.2	C36A—C35A—N2A	118.7 (2)
C71A—C7A—H7A	107.2	C34A—C35A—N2A	119.2 (2)
C5A—C7A—H7A	107.2	C36B—C35B—C34B	122.09 (18)
C8B—C7B—C71B	117.92 (14)	C36B—C35B—N2B	119.8 (2)
C8B—C7B—C5B	102.36 (13)	C34B—C35B—N2B	118.10 (19)
C71B—C7B—C5B	114.82 (14)	C35A—C36A—C37A	118.85 (19)
C8B—C7B—H7B	107.0	C35A—C36A—H36A	120.6
C71B—C7B—H7B	107.0	C37A—C36A—H36A	120.6
C5B—C7B—H7B	107.0	C35B—C36B—C37B	119.07 (19)
N3A—C8A—C9A	105.06 (15)	C35B—C36B—H36B	120.5
N3A—C8A—C7A	100.15 (13)	C37B—C36B—H36B	120.5
C9A—C8A—C7A	119.57 (16)	C36A—C37A—C32A	121.1 (2)
N3A—C8A—H8A	110.4	C36A—C37A—H37A	119.4
C9A—C8A—H8A	110.4	C32A—C37A—H37A	119.4
C7A—C8A—H8A	110.4	C36B—C37B—C32B	120.98 (18)
N3B—C8B—C9B	104.42 (14)	C36B—C37B—H37B	119.5
N3B—C8B—C7B	100.06 (12)	C32B—C37B—H37B	119.5
C9B—C8B—C7B	119.34 (15)	C72A—C71A—C76A	118.22 (17)
N3B—C8B—H8B	110.7	C72A—C71A—C7A	122.83 (16)
C9B—C8B—H8B	110.7	C76A—C71A—C7A	118.94 (15)
C7B—C8B—H8B	110.7	C76B—C71B—C72B	117.98 (16)
C8A—C9A—S1A	104.85 (14)	C76B—C71B—C7B	122.36 (16)
C8A—C9A—H9A1	110.8	C72B—C71B—C7B	119.64 (15)
S1A—C9A—H9A1	110.8	C73A—C72A—C71A	120.48 (19)
C8A—C9A—H9A2	110.8	C73A—C72A—H72A	119.8
S1A—C9A—H9A2	110.8	C71A—C72A—H72A	119.8
H9A1—C9A—H9A2	108.9	C73B—C72B—C71B	121.05 (18)
C8B—C9B—S1B	104.97 (12)	C73B—C72B—H72B	119.5
C8B—C9B—H9B1	110.8	C71B—C72B—H72B	119.5
S1B—C9B—H9B1	110.8	C74A—C73A—C72A	119.84 (19)
C8B—C9B—H9B2	110.8	C74A—C73A—H73A	120.1
S1B—C9B—H9B2	110.8	C72A—C73A—H73A	120.1
H9B1—C9B—H9B2	108.8	C74B—C73B—C72B	119.02 (19)
N3A—C10A—S1A	103.77 (13)	C74B—C73B—H73B	120.5
N3A—C10A—H10A	111.0	C72B—C73B—H73B	120.5
S1A—C10A—H10A	111.0	C73A—C74A—C75A	121.34 (18)
N3A—C10A—H10B	111.0	C73A—C74A—N4A	119.5 (2)
S1A—C10A—H10B	111.0	C75A—C74A—N4A	119.2 (2)
H10A—C10A—H10B	109.0	C75B—C74B—C73B	121.55 (18)
N3B—C10B—S1B	104.06 (12)	C75B—C74B—N4B	118.8 (2)
N3B—C10B—H10C	110.9	C73B—C74B—N4B	119.6 (2)
S1B—C10B—H10C	110.9	C74A—C75A—C76A	118.6 (2)
N3B—C10B—H10D	110.9	C74A—C75A—H75A	120.7
S1B—C10B—H10D	110.9	C76A—C75A—H75A	120.7
H10C—C10B—H10D	109.0	C74B—C75B—C76B	119.11 (19)
N3A—C11A—C19A	111.57 (14)	C74B—C75B—H75B	120.4
N3A—C11A—C12A	113.36 (13)	C76B—C75B—H75B	120.4
C19A—C11A—C12A	101.75 (13)	C75A—C76A—C71A	121.53 (18)
N3A—C11A—C5A	101.21 (12)	C75A—C76A—H76A	119.2
C19A—C11A—C5A	117.74 (13)	C71A—C76A—H76A	119.2
C12A—C11A—C5A	111.73 (13)	C75B—C76B—C71B	121.29 (19)
N3B—C11B—C19B	111.98 (13)	C75B—C76B—H76B	119.4
N3B—C11B—C12B	113.71 (14)	C71B—C76B—H76B	119.4
C19B—C11B—C12B	101.67 (13)	C21B—C22B—C17B	120.7 (2)
N3B—C11B—C5B	101.68 (12)	C21B—C22B—H22B	119.7
C19B—C11B—C5B	115.73 (13)	C17B—C22B—H22B	119.7
C12B—C11B—C5B	112.59 (13)	C2A—N1A—C6A	111.74 (14)
O4A—C12A—C13A	127.13 (17)	C2A—N1A—C1A	111.07 (14)
O4A—C12A—C11A	123.82 (16)	C6A—N1A—C1A	113.39 (14)
C13A—C12A—C11A	108.54 (15)	C6B—N1B—C1B	113.20 (14)
O4B—C12B—C13B	127.88 (18)	C6B—N1B—C2B	112.52 (14)
O4B—C12B—C11B	123.96 (17)	C1B—N1B—C2B	110.65 (15)
C13B—C12B—C11B	107.59 (16)	O3A—N2A—O2A	123.5 (2)
C14A—C13A—C18A	120.52 (19)	O3A—N2A—C35A	118.6 (2)
C14A—C13A—C12A	132.42 (19)	O2A—N2A—C35A	117.8 (2)
C18A—C13A—C12A	107.05 (16)	O3B—N2B—O2B	123.6 (2)
C14B—C13B—C18B	119.7 (2)	O3B—N2B—C35B	118.4 (2)
C14B—C13B—C12B	132.8 (2)	O2B—N2B—C35B	117.8 (2)
C18B—C13B—C12B	107.44 (16)	C10A—N3A—C8A	110.40 (14)
C13A—C14A—C15A	117.8 (2)	C10A—N3A—C11A	120.56 (14)
C13A—C14A—H14A	121.1	C8A—N3A—C11A	108.50 (13)
C15A—C14A—H14A	121.1	C10B—N3B—C11B	120.76 (14)
C13B—C14B—C15B	117.5 (3)	C10B—N3B—C8B	110.51 (13)
C13B—C14B—H14B	121.2	C11B—N3B—C8B	108.83 (13)
C15B—C14B—H14B	121.2	O5A—N4A—O6A	123.0 (2)
C16A—C15A—C14A	122.4 (2)	O5A—N4A—C74A	118.9 (2)
C16A—C15A—H15A	118.8	O6A—N4A—C74A	118.1 (2)
C14A—C15A—H15A	118.8	O6B—N4B—O5B	123.4 (2)
C16B—C15B—C14B	122.8 (2)	O6B—N4B—C74B	119.2 (2)
C16B—C15B—H15B	118.6	O5B—N4B—C74B	117.4 (2)
C14B—C15B—H15B	118.6	C10A—S1A—C9A	93.73 (9)
C15A—C16A—C17A	120.7 (2)	C9B—S1B—C10B	93.57 (8)
			
N1A—C2A—C3A—C31A	−156.81 (17)	C13B—C18B—C19B—C20B	175.10 (17)
N1A—C2A—C3A—C4A	21.9 (2)	C17B—C18B—C19B—C20B	−2.8 (3)
N1B—C2B—C3B—C31B	157.16 (17)	C13B—C18B—C19B—C11B	−5.0 (2)
N1B—C2B—C3B—C4B	−22.0 (2)	C17B—C18B—C19B—C11B	177.08 (17)
C31A—C3A—C4A—O1A	−21.9 (2)	N3B—C11B—C19B—C20B	−52.4 (2)
C2A—C3A—C4A—O1A	159.25 (16)	C12B—C11B—C19B—C20B	−174.10 (19)
C31A—C3A—C4A—C5A	157.53 (15)	C5B—C11B—C19B—C20B	63.5 (2)
C2A—C3A—C4A—C5A	−21.3 (2)	N3B—C11B—C19B—C18B	127.78 (15)
C31B—C3B—C4B—O1B	22.4 (2)	C12B—C11B—C19B—C18B	6.03 (18)
C2B—C3B—C4B—O1B	−158.36 (17)	C5B—C11B—C19B—C18B	−116.32 (16)
C31B—C3B—C4B—C5B	−157.45 (15)	C18A—C19A—C20A—C21A	−4.0 (3)
C2B—C3B—C4B—C5B	21.8 (2)	C11A—C19A—C20A—C21A	177.93 (18)
O1A—C4A—C5A—C6A	−138.97 (16)	C18B—C19B—C20B—C21B	4.4 (3)
C3A—C4A—C5A—C6A	41.59 (18)	C11B—C19B—C20B—C21B	−175.47 (18)
O1A—C4A—C5A—C7A	−13.6 (2)	C19A—C20A—C21A—C22A	2.6 (3)
C3A—C4A—C5A—C7A	166.97 (13)	C19B—C20B—C21B—C22B	−2.5 (3)
O1A—C4A—C5A—C11A	101.69 (18)	C20A—C21A—C22A—C17A	0.7 (4)
C3A—C4A—C5A—C11A	−77.74 (17)	C18A—C17A—C22A—C21A	−2.3 (3)
O1B—C4B—C5B—C6B	138.27 (16)	C16A—C17A—C22A—C21A	175.3 (2)
C3B—C4B—C5B—C6B	−41.90 (18)	C4A—C3A—C31A—C32A	−179.26 (17)
O1B—C4B—C5B—C7B	13.5 (2)	C2A—C3A—C31A—C32A	−0.5 (3)
C3B—C4B—C5B—C7B	−166.67 (14)	C4B—C3B—C31B—C32B	179.15 (18)
O1B—C4B—C5B—C11B	−101.37 (18)	C2B—C3B—C31B—C32B	0.0 (3)
C3B—C4B—C5B—C11B	78.47 (17)	C3A—C31A—C32A—C37A	160.6 (2)
C4A—C5A—C6A—N1A	−65.84 (17)	C3A—C31A—C32A—C33A	−22.4 (3)
C7A—C5A—C6A—N1A	168.66 (13)	C3B—C31B—C32B—C37B	18.9 (3)
C11A—C5A—C6A—N1A	52.00 (17)	C3B—C31B—C32B—C33B	−163.6 (2)
C4B—C5B—C6B—N1B	65.10 (17)	C37A—C32A—C33A—C34A	−0.3 (3)
C7B—C5B—C6B—N1B	−170.44 (13)	C31A—C32A—C33A—C34A	−177.38 (18)
C11B—C5B—C6B—N1B	−53.86 (17)	C37B—C32B—C33B—C34B	0.8 (3)
C4A—C5A—C7A—C8A	139.87 (14)	C31B—C32B—C33B—C34B	−176.9 (2)
C6A—C5A—C7A—C8A	−98.37 (16)	C32A—C33A—C34A—C35A	1.2 (3)
C11A—C5A—C7A—C8A	22.07 (16)	C32B—C33B—C34B—C35B	−0.9 (3)
C4A—C5A—C7A—C71A	−91.65 (17)	C33A—C34A—C35A—C36A	−1.0 (3)
C6A—C5A—C7A—C71A	30.1 (2)	C33A—C34A—C35A—N2A	178.38 (19)
C11A—C5A—C7A—C71A	150.56 (14)	C33B—C34B—C35B—C36B	0.2 (3)
C4B—C5B—C7B—C8B	−142.61 (14)	C33B—C34B—C35B—N2B	179.7 (2)
C6B—C5B—C7B—C8B	96.09 (16)	C34A—C35A—C36A—C37A	−0.1 (4)
C11B—C5B—C7B—C8B	−24.71 (15)	N2A—C35A—C36A—C37A	−179.5 (2)
C4B—C5B—C7B—C71B	88.38 (17)	C34B—C35B—C36B—C37B	0.5 (3)
C6B—C5B—C7B—C71B	−32.91 (19)	N2B—C35B—C36B—C37B	−179.00 (18)
C11B—C5B—C7B—C71B	−153.71 (14)	C35A—C36A—C37A—C32A	1.1 (4)
C71A—C7A—C8A—N3A	−168.08 (14)	C33A—C32A—C37A—C36A	−0.9 (3)
C5A—C7A—C8A—N3A	−41.87 (16)	C31A—C32A—C37A—C36A	176.4 (2)
C71A—C7A—C8A—C9A	78.0 (2)	C35B—C36B—C37B—C32B	−0.6 (3)
C5A—C7A—C8A—C9A	−155.76 (16)	C33B—C32B—C37B—C36B	0.0 (3)
C71B—C7B—C8B—N3B	170.29 (14)	C31B—C32B—C37B—C36B	177.42 (18)
C5B—C7B—C8B—N3B	43.25 (15)	C8A—C7A—C71A—C72A	14.7 (2)
C71B—C7B—C8B—C9B	−76.8 (2)	C5A—C7A—C71A—C72A	−106.05 (19)
C5B—C7B—C8B—C9B	156.20 (15)	C8A—C7A—C71A—C76A	−166.37 (16)
N3A—C8A—C9A—S1A	36.67 (17)	C5A—C7A—C71A—C76A	72.9 (2)
C7A—C8A—C9A—S1A	147.91 (14)	C8B—C7B—C71B—C76B	−12.5 (2)
N3B—C8B—C9B—S1B	−37.70 (16)	C5B—C7B—C71B—C76B	108.31 (19)
C7B—C8B—C9B—S1B	−148.29 (13)	C8B—C7B—C71B—C72B	165.65 (16)
C4A—C5A—C11A—N3A	−115.31 (14)	C5B—C7B—C71B—C72B	−73.6 (2)
C6A—C5A—C11A—N3A	127.98 (13)	C76A—C71A—C72A—C73A	−0.3 (3)
C7A—C5A—C11A—N3A	5.87 (15)	C7A—C71A—C72A—C73A	178.71 (17)
C4A—C5A—C11A—C19A	6.54 (19)	C76B—C71B—C72B—C73B	−0.9 (3)
C6A—C5A—C11A—C19A	−110.18 (16)	C7B—C71B—C72B—C73B	−179.14 (16)
C7A—C5A—C11A—C19A	127.71 (15)	C71A—C72A—C73A—C74A	−1.0 (3)
C4A—C5A—C11A—C12A	123.75 (15)	C71B—C72B—C73B—C74B	0.5 (3)
C6A—C5A—C11A—C12A	7.03 (18)	C72A—C73A—C74A—C75A	1.1 (3)
C7A—C5A—C11A—C12A	−115.08 (14)	C72A—C73A—C74A—N4A	−179.73 (18)
C4B—C5B—C11B—N3B	117.27 (14)	C72B—C73B—C74B—C75B	0.4 (3)
C6B—C5B—C11B—N3B	−125.09 (13)	C72B—C73B—C74B—N4B	178.22 (17)
C7B—C5B—C11B—N3B	−3.05 (15)	C73A—C74A—C75A—C76A	0.0 (3)
C4B—C5B—C11B—C19B	−4.32 (18)	N4A—C74A—C75A—C76A	−179.13 (17)
C6B—C5B—C11B—C19B	113.32 (15)	C73B—C74B—C75B—C76B	−0.8 (3)
C7B—C5B—C11B—C19B	−124.64 (14)	N4B—C74B—C75B—C76B	−178.62 (18)
C4B—C5B—C11B—C12B	−120.67 (15)	C74A—C75A—C76A—C71A	−1.3 (3)
C6B—C5B—C11B—C12B	−3.02 (18)	C72A—C71A—C76A—C75A	1.4 (3)
C7B—C5B—C11B—C12B	119.01 (14)	C7A—C71A—C76A—C75A	−177.58 (16)
N3A—C11A—C12A—O4A	−49.7 (2)	C74B—C75B—C76B—C71B	0.3 (3)
C19A—C11A—C12A—O4A	−169.64 (17)	C72B—C71B—C76B—C75B	0.5 (3)
C5A—C11A—C12A—O4A	63.9 (2)	C7B—C71B—C76B—C75B	178.70 (17)
N3A—C11A—C12A—C13A	122.68 (16)	C20B—C21B—C22B—C17B	−1.3 (4)
C19A—C11A—C12A—C13A	2.76 (18)	C16B—C17B—C22B—C21B	−175.7 (2)
C5A—C11A—C12A—C13A	−123.73 (15)	C18B—C17B—C22B—C21B	2.9 (3)
N3B—C11B—C12B—O4B	46.3 (2)	C3A—C2A—N1A—C6A	−46.7 (2)
C19B—C11B—C12B—O4B	166.82 (18)	C3A—C2A—N1A—C1A	−174.37 (15)
C5B—C11B—C12B—O4B	−68.7 (2)	C5A—C6A—N1A—C2A	70.98 (17)
N3B—C11B—C12B—C13B	−125.69 (16)	C5A—C6A—N1A—C1A	−162.57 (15)
C19B—C11B—C12B—C13B	−5.15 (18)	C5B—C6B—N1B—C1B	163.39 (16)
C5B—C11B—C12B—C13B	119.33 (15)	C5B—C6B—N1B—C2B	−70.25 (18)
O4A—C12A—C13A—C14A	−7.6 (4)	C3B—C2B—N1B—C6B	46.4 (2)
C11A—C12A—C13A—C14A	−179.7 (2)	C3B—C2B—N1B—C1B	174.13 (16)
O4A—C12A—C13A—C18A	170.98 (18)	C36A—C35A—N2A—O3A	−163.2 (2)
C11A—C12A—C13A—C18A	−1.1 (2)	C34A—C35A—N2A—O3A	17.4 (3)
O4B—C12B—C13B—C14B	9.5 (4)	C36A—C35A—N2A—O2A	19.0 (3)
C11B—C12B—C13B—C14B	−178.9 (2)	C34A—C35A—N2A—O2A	−160.4 (2)
O4B—C12B—C13B—C18B	−168.95 (19)	C36B—C35B—N2B—O3B	−15.8 (3)
C11B—C12B—C13B—C18B	2.6 (2)	C34B—C35B—N2B—O3B	164.7 (2)
C18A—C13A—C14A—C15A	0.3 (3)	C36B—C35B—N2B—O2B	161.2 (2)
C12A—C13A—C14A—C15A	178.7 (2)	C34B—C35B—N2B—O2B	−18.4 (3)
C18B—C13B—C14B—C15B	−0.6 (3)	S1A—C10A—N3A—C8A	40.47 (17)
C12B—C13B—C14B—C15B	−178.9 (2)	S1A—C10A—N3A—C11A	168.26 (12)
C13A—C14A—C15A—C16A	−0.4 (4)	C9A—C8A—N3A—C10A	−52.04 (19)
C13B—C14B—C15B—C16B	−0.2 (4)	C7A—C8A—N3A—C10A	−176.61 (14)
C14A—C15A—C16A—C17A	0.6 (4)	C9A—C8A—N3A—C11A	173.81 (15)
C14B—C15B—C16B—C17B	1.2 (5)	C7A—C8A—N3A—C11A	49.25 (16)
C15A—C16A—C17A—C18A	−0.5 (3)	C19A—C11A—N3A—C10A	71.2 (2)
C15A—C16A—C17A—C22A	−178.1 (2)	C12A—C11A—N3A—C10A	−43.0 (2)
C15B—C16B—C17B—C22B	177.5 (3)	C5A—C11A—N3A—C10A	−162.77 (15)
C15B—C16B—C17B—C18B	−1.1 (4)	C19A—C11A—N3A—C8A	−160.18 (14)
C16A—C17A—C18A—C13A	0.4 (3)	C12A—C11A—N3A—C8A	85.66 (17)
C22A—C17A—C18A—C13A	178.31 (19)	C5A—C11A—N3A—C8A	−34.13 (16)
C16A—C17A—C18A—C19A	−177.18 (19)	S1B—C10B—N3B—C11B	−168.56 (12)
C22A—C17A—C18A—C19A	0.8 (3)	S1B—C10B—N3B—C8B	−39.99 (17)
C14A—C13A—C18A—C17A	−0.3 (3)	C19B—C11B—N3B—C10B	−74.39 (19)
C12A—C13A—C18A—C17A	−179.05 (17)	C12B—C11B—N3B—C10B	40.2 (2)
C14A—C13A—C18A—C19A	177.52 (18)	C5B—C11B—N3B—C10B	161.46 (14)
C12A—C13A—C18A—C19A	−1.3 (2)	C19B—C11B—N3B—C8B	156.29 (13)
C14B—C13B—C18B—C19B	−177.28 (19)	C12B—C11B—N3B—C8B	−89.15 (17)
C12B—C13B—C18B—C19B	1.4 (2)	C5B—C11B—N3B—C8B	32.14 (16)
C14B—C13B—C18B—C17B	0.6 (3)	C9B—C8B—N3B—C10B	52.20 (18)
C12B—C13B—C18B—C17B	179.30 (18)	C7B—C8B—N3B—C10B	176.23 (14)
C22B—C17B—C18B—C13B	−178.52 (19)	C9B—C8B—N3B—C11B	−173.01 (14)
C16B—C17B—C18B—C13B	0.3 (3)	C7B—C8B—N3B—C11B	−48.99 (16)
C22B—C17B—C18B—C19B	−0.8 (3)	C73A—C74A—N4A—O5A	−8.3 (3)
C16B—C17B—C18B—C19B	177.97 (19)	C75A—C74A—N4A—O5A	170.9 (2)
C17A—C18A—C19A—C20A	2.5 (3)	C73A—C74A—N4A—O6A	172.5 (2)
C13A—C18A—C19A—C20A	−175.29 (16)	C75A—C74A—N4A—O6A	−8.3 (3)
C17A—C18A—C19A—C11A	−179.08 (17)	C75B—C74B—N4B—O6B	3.3 (3)
C13A—C18A—C19A—C11A	3.2 (2)	C73B—C74B—N4B—O6B	−174.6 (2)
N3A—C11A—C19A—C20A	53.5 (2)	C75B—C74B—N4B—O5B	−177.3 (2)
C12A—C11A—C19A—C20A	174.70 (19)	C73B—C74B—N4B—O5B	4.8 (3)
C5A—C11A—C19A—C20A	−62.8 (3)	N3A—C10A—S1A—C9A	−14.43 (15)
N3A—C11A—C19A—C18A	−124.66 (15)	C8A—C9A—S1A—C10A	−12.82 (16)
C12A—C11A—C19A—C18A	−3.49 (18)	C8B—C9B—S1B—C10B	14.16 (14)
C5A—C11A—C19A—C18A	118.97 (16)	N3B—C10B—S1B—C9B	13.34 (14)

### Hydrogen-bond geometry (Å, º)

**Table:**

*D*—H···*A*	*D*—H	H···*A*	*D*···*A*	*D*—H···*A*
C8*B*—H8*B*···O4*A*	0.98	2.47	3.270 (2)	139
C72*A*—H72*A*···O4*B*	0.93	2.55	3.461 (2)	167
C76*B*—H76*B*···O4*A*	0.93	2.30	3.222 (2)	174
C15*B*—H15*B*···O3*B*^i^	0.93	2.50	3.343 (3)	151

Symmetry code: (i) −*x*, −*y*+1, −*z*.

## References

[bb1] Bruker (2004). *APEX2* and *SAINT* Bruker AXS Inc., Madison, Wisconsin, USA.

[bb2] Sheldrick, G. M. (1996). *SADABS*, University of Göttingen, Germany.

[bb3] Sheldrick, G. M. (2008). *Acta Cryst.* A**64**, 112–122. 10.1107/S0108767307043930 18156677

[bb4] Spek, A. L. (2009). *Acta Cryst.* D**65**, 148–155. 10.1107/S090744490804362X PMC2631630 19171970

[bb5] Suresh, J., Nagalakshmi, R. A., Sivakumar, S., Kumar, R. R. & Lakshman, P. L. N. (2013). *Acta Cryst.* E**69**, o140–o141. 10.1107/S1600536812051094 PMC3588349 23476397

